# One-Stage Immediate Breast Reconstruction: A Concise Review

**DOI:** 10.1155/2017/6486859

**Published:** 2017-10-02

**Authors:** Nicolò Bertozzi, Marianna Pesce, Pierluigi Santi, Edoardo Raposio

**Affiliations:** ^1^Department of Medicine and Surgery, Plastic Surgery Division, University of Parma, Parma, Italy; ^2^Cutaneous, Mini-Invasive, Regenerative and Plastic Surgery Unit, Parma University Hospital, Parma, Italy; ^3^Department of Surgical Sciences and Integrated Diagnostics, University of Genoa, Genoa, Italy; ^4^Plastic Surgery Department, IRCCS San Martino University Hospital, National Institute for Cancer Research Genoa, Genoa, Italy

## Abstract

**Background:**

One-stage direct-to-implant immediate breast reconstruction (IBR) is performed simultaneously with breast cancer resection. We explored indications, techniques, and outcomes of IBR to determine its feasibility, safety, and effectiveness.

**Material and Methods:**

We reviewed the available literature on one-stage direct-to-implant IBR, with or without acellular dermal matrix (ADM), synthetic mesh, or autologous fat grafting. We analyzed the indications, preoperative work-up, surgical technique, postoperative care, outcomes, and complications.

**Results:**

IBR is indicated for small-to-medium nonptotic breasts and contraindicated in patients who require or have undergone radiotherapy, due to unacceptably high complications rates. Only patients with thick, well-vascularized mastectomy flaps are IBR candidates. Expandable implants should be used for ptotic breasts, while anatomical shaped implants should be used to reconstruct small-to-medium nonptotic breasts. ADMs can be used to cover the implant during IBR and avoid muscle elevation, thereby minimizing postoperative pain. Flap necrosis, reoperation, and implant loss are more common with IBR than conventional two-staged reconstruction, but IBR has advantages such as lack of secondary surgery, faster recovery, and better quality of life.

**Conclusions:**

IBR has good outcomes and patient-satisfaction rates. With ADM use, a shift from conventional reconstruction to IBR has occurred. Drawbacks of IBR can be overcome by careful patient selection.

## 1. Background

Since skin- and nipple-sparing mastectomies have proven to be oncologically safe, an increasing number of patients with invasive breast cancer undergo breast reconstruction [1, 2]. Indeed, for women who have undergone a mastectomy, breast reconstruction provides psychosocial as well as aesthetic benefits [3–5]. Breast reconstruction can be either allogeneic (implant-based), autologous (locoregional flap, free flap), or a combination of both. Reconstruction can be performed simultaneously with mastectomy as a one- or two-stage procedure, or it can be delayed and performed as a two-stage procedure.

Implant-based reconstructions account for almost 65% of all breast reconstructions in the USA [6, 7]. This type of reconstruction is considered safe, cost effective, and reliable; furthermore, it can be performed in women with a wide variety of comorbid conditions [8, 9]. One-stage immediate breast reconstruction (IBR) is a method to reconstruct a definitive breast mound at the time of oncologic resection without the need for tissue expansion or tissue expander/implant exchange. However, the likelihood of requiring secondary procedure (e.g., scar revision, autologous fat grafting, nipple-areola complex (NAC) reconstruction, and matching surgery to the contralateral breast) is not lower after one-stage IBR than after two-stage implant-based IBR [10]. Given the fewer hospital accesses required, IBR may be convenient for both patients and the healthcare system [10], and this partially explains the increasing number of IBRs performed [11, 12]. The recent introduction of acellular dermal matrices (ADMs) and synthetic meshes has also widened the indications for IBR [13–15].

Advocates of IBR highlight its advantages, which include elimination of the expander-to-implant exchange, fast recovery, better lower pole definition, and lower costs [16–18]. However, several studies have reported higher rates of complications and implant loss with direct-to-implant IBR [19–22].

Given the conflicting reports, we aimed to identify from the current literature the indications, techniques, and outcomes of IBR in order to help surgeons in choosing and performing the most suitable breast reconstruction for each patient. We reviewed the available literature on one-stage direct-to-implant IBR, with or without acellular dermal matrix (ADM), synthetic mesh, or autologous fat grafting. We analyzed the indications, preoperative work-up, surgical technique, postoperative care, outcomes, and complications, including the need for secondary procedures, of one-stage direct-to-implant IBR.

## 2. General Considerations

Breast cancer is the most common malignancy in women [23]. In 2011, it was estimated that nearly 230,000 women were diagnosed with invasive breast cancer in the USA alone [24]; 79% of the 96,277 patients who underwent breast reconstructions underwent alloplastic-based breast reconstruction [25]. Following advances in screening tests and molecular genetics, many young women (aged <45 years) with inherited predisposition genes for breast cancer are choosing to undergo bilateral prophylactic mastectomy [26]. Even women with early-stage breast cancer suitable for breast-conserving surgery may choose to undergo therapeutic mastectomy and contralateral prophylactic mastectomy [27]. These young women may obtain more pleasing cosmetic outcomes by means of autologous reconstruction but may not agree to undergo major surgery, with more scar formation at the donor site. Alloplastic breast reconstruction can ensure satisfactory cosmetic outcomes in such patients with far less-invasive surgery. Furthermore, more than 50% of breast cancer cases occur in elderly women who may opt for a less-invasive, one-stage procedure associated with early discharge, rapid recovery, and a prompt return to everyday life [28].

## 3. Acellular Dermal Matrix and Synthetic Mesh

Dieterich et al. [14] and Salzberg [29] first reported the use of acellular dermal matrices (ADMs) for breast reconstruction. This strategy gradually spread among reconstructive surgeons, even though early studies reported higher complication rates with ADMs than with conventional breast reconstruction [30, 31]. However, recent works have highlighted a reduction in complication rates, as a result of the increased familiarity of plastic surgeons with the use of ADMs [32–34]. Currently, members of the American Society of Plastic Surgeons use ADMs in more than 50% of their breast reconstructions [35].

ADMs are made of extracellular matrix structures and basement membrane complexes of either of fetal bovine, porcine, or human cadaver origin [36]. ADMs lack immunogenic epitopes and are therefore revascularized, recellularized, and integrated into host tissue with no evidence of encapsulation, resorption, or contracture [37–41]. In breast reconstructions with submuscular implants, ADMs can be used as pectoral expanders to cover the inferolateral pole of the implant and prevent the need for elevation of the surrounding muscle, thus reducing postoperative pain [18, 42–44]. ADMs can also be used as an internal bra to completely cover the implant during subcutaneous breast reconstruction, anchoring the implant to the chest wall and providing an additional layer of tissue supports, thereby relieving the pressure on the mastectomy flaps [45–47].

Synthetic meshes were recently introduced as an alternative to ADMs in alloplastic breast reconstruction and have shown promising results [15, 47–50]. Synthetic meshes are safe and have aesthetic benefits similar to those obtained with ADMs (improved inframammary fold definition, enhanced lower pole shape and projection) without the drawbacks of high cost and local policy restrictions.

Some authors have proposed that ADMs be substituted with autologous dermal grafts harvested at the time of mastectomy as a horizontally oriented ellipse in the lower abdomen if a preexisting scar is present or from the contralateral breast if a reductive mastoplasty is planned [51–53]. In a prospective study, Lynch et al. [51] compared the outcomes of dermal autograft- and ADM-assisted breast reconstructions. They found that the major and minor complication rates as well as total costs were higher in the ADM group, while cosmetic outcomes did not significantly differ between the ADM and autograft groups. Moreover, histological analysis showed higher integration of the autograft into the surrounding tissue, with extensive revascularization and vessel ingrowth.

## 4. Immediate Breast Reconstruction 

### 4.1. Indications

Direct-to-implant IBR aims to create a naturally appearing breast mound in a single-stage surgery without harnessing the mastectomy flap blood supply. Preoperative and intraoperative evaluations commonly guide surgeons' decision to perform IBR. Young, thin, and athletic women with small-to-medium, nonptotic breasts are best suited for IBR as well as SSM [54]. However, skin-reducing mastectomy has broadened the indications of IBR to patients with a very large breast skin envelope [55]. Nevertheless, patients undergoing unilateral reconstruction must be aware that they may eventually need to undergo contralateral matching surgery [56].

ADM-assisted IBR enables the reconstruction of breasts with varying degrees of ptosis [57]. In patients with a discrepancy between muscular coverage and native breast skin-flap coverage, ADMs can be used to lengthen the muscular tissue plane to match the overlying skin envelope. Thus, the reconstructed breast may be left with a natural pseudo-ptosis, a well-defined inframammary fold, and enhanced inferior pole projection, which better match the contralateral native breast [58]. The estimated mastectomy weight should not exceed 600 g [58]. Characteristics of the breast soft tissue, skin elasticity of the trunk and overall body habitus are commonly evaluated preoperative parameters [10].

As with expander-based breast reconstruction, direct-to-implant IBR is reported to have higher complication rates in patients who are scheduled to undergo adjuvant radiotherapy or who have a history of local irradiation; hence, the indication of IBR in such patients remains controversial [59]. Unacceptably high complication rates (i.e., capsular contracture and infection) have been reported with implant-based reconstruction followed by radiation therapy [17, 18, 60], and delayed autologous tissue reconstruction after mastectomy radiation therapy is generally regarded as the best approach [61]. Nevertheless, increased surgical expertise and improved target breast irradiation can pave the way to a safer implant-based breast reconstruction even when adjuvant radiotherapy is required [62–66]. Conflicting reports are available regarding the use of ADMs when adjuvant radiation therapy is required [67–71].

Well-vascularized mastectomy skin flaps with an at least 1 cm thick subcutaneous layer are unanimously considered to be essential to achieve successful outcomes [10, 72]. The intraoperative judgment of the surgeon is recognized as the single most important factor influencing the success of direct-to-implant IBR [17, 34, 73, 74]. Indeed, only patients with good-quality mastectomy flaps (thick and well-vascularized) should be candidates for IBR to minimize the chances of mastectomy flap necrosis [58]. The advent of intraoperative objective assessment tools such as real-time perfusion mapping assisted by SPY® (Novadaq Technologies Inc., Bonita Springs, FL, USA) has helped surgeons in this key decision-making stage [75].

### 4.2. Preoperative Planning

The oncologic surgeon and the plastic surgeon should perform the preoperative evaluation together. Breast cancer localization, dimensions, and nipple-areola involvement must be cautiously evaluated to decide between skin-sparing mastectomy and nipple-sparing mastectomy. Preoperative markings should favorably locate the mastectomy scar, while preserving the required skin envelope. The borders of the breast must be marked with the patient standing upright, paying particular care in the marking of the inframammary fold. The upper-pole border should match the level of the contralateral breast, which can be delineated by gently compressing the contralateral breast against the chest wall. Nipple-to-sternal notch distance, areola-to-inframammary fold distance, and breast width are measured at this stage. Skin quality, elasticity, and thickness must also be carefully assessed. When contralateral matching surgery is planned, preoperative markings are also drawn on the contralateral breast, depending on the procedure required. At this stage, surgeons should also bear in mind the type and dimensions of the implant required [13, 76].

When performing unilateral reconstruction in elderly women with ptotic breasts, permanent expandable implants provide good long-term cosmetic and oncologic outcomes [10, 77]. Becker 25 and 50 (round) and Becker 35 (shaped) implants (Mentor, Johnson & Johnson Medical Ltd., Wokingham, Berkshire, UK) as well as anatomical Style 150 implants (Allergan Inc., Irvine, CA, USA) are commonly used permanent expandable biodimensional implants. These implants differ not only in terms of the silicone elastomer used but also in terms of their capacity; the Becker series implants can be overinflated for at least 1 month and subsequently deflated. This strategy allows matching to the contralateral perimenopausal breast by creating a pseudo-ptotic effect, lowering the position of the NAC, and lessening the final projection [10, 77, 78].

Anatomic, shaped, silicone-filled implants are best suited for recreating small-to-mild nonptotic breasts or when ADM slings are required [13]. Indeed, silicone-filled implants tend to lie flat against the chest wall; hence, anatomic shapes provide appropriate volume restoration without excessive superior-pole fullness. Even round implants can achieve good results; however, there is a major risk of developing unpleasing superior border step-off. Finally, the base diameter of the implant should match that of the contralateral breast, for both round and anatomically shaped implants [13]. Given the complexity of this decision-making stage, surgical planning and virtual simulator systems have been devised to train surgeons outside of the “apprenticeship model” [79–81].

### 4.3. Surgical Technique

The mastectomy can be a simple skin-sparing or skin-reducing NAC-sparing mastectomy [82, 83]. The mastectomy should be conducted in the space between the subcutaneous adipose tissue and the glandular parenchyma so as not to damage the subdermal plexus of the mastectomy flaps and jeopardize their blood supply [47]. The inframammary fold is a crucial landmark of the breast and must be conserved during mastectomy [84, 85]. If damaged, it has to be restored using 4-5 braided silk or Vicryl® sutures [56].

Once the mastectomy is completed, the surgeon should assess skin-flap viability by evaluating skin-flap color, capillary refill, temperature, turgor, and dermal bleeding [86]. Fluorescein angiography and laser-assisted indocyanine green angiography can also be used to objectively assess the vitality of the mastectomy flaps [87, 88]. If the flaps are deemed viable, direct-to-implant IBR can be performed.

At this stage, the reconstructive surgeon has various options. An implant pocket can be created in either the submuscular (partial or complete) or subcutaneous plane, and ADMs, synthetic meshes, or dermal autografts can be employed. Advocates of muscular coverage of implants aim to maximize vascularity and prevent contact between the implant and the overlying mastectomy incision [56, 85]. With the patient in a supine position and the ipsilateral upper arm adducted at 60°, the pectoralis major muscle is dissected from its thoracic and sternal attachments, till the second rib [85]. A subpectoral pocket is superiorly dissected in a relatively avascular plane, following the preoperative markings. Raising either the serratus anterior muscle completely or its lower slips only (to reduce postoperative pain) can achieve complete muscular coverage of the implants [89].

A partial submuscular pocket can also be created by elevating the serratus anterior muscle in a plane within the muscle along with its overlying fascia, leaving the rib cage covered by a portion of the muscle [82]. Inferiorly, elevating the fascia of the anterior rectus muscle completes the pocket. When a skin-reducing mastectomy is performed, the dermal-adipose inferior flap is used for coverage of the lower pole [90]. The submuscular pocket can also be completed inferiorly with a rectangular 6 cm × 16 cm piece of ADM, synthetic mesh, or dermal autograft. The chosen graft is sutured to the inferior and lateral chest walls and to the inferior portion of the pectoralis muscle, acting as a hammock for the lower pole of the chosen implant. ADMs, synthetic meshes or dermal autografts should be accurately tailored so as to prevent seromas, which may result from discrepancies between muscular coverage and the native breast skin envelope, contour irregularities, or dead space [57]. The adequacy of the position of the inframammary fold and shape of the breast mound are assessed intraoperatively by placing the patient in a sitting position. The definitive implant can also be positioned in the subcutaneous pocket resulting from the mastectomy.

The implant is completely wrapped by an ADM or synthetic mesh so as to suture together the membrane. The ADM-wrapped implant can be positioned into the subcutaneous pocket and secured in place to the underlying muscles by means of apical, medial, and lateral absorbable stitches [58].

Once the pocket has been created, it must be thoroughly irrigated, and accurate hemostasis must be performed. Various irrigant solutions have been proposed to prevent subclinical pocket infection, which is regarded as a possible etiology of capsular contracture [56]. The triple antibiotic of Adams (comprising 80 mg gentamicin, 1 g cefazolin, and 50,000 U bacitracin [or equivalent vancomycin], diluted in 500 mL normal saline), single antibiotic solution, diluted povidone-iodine, and normal saline are the most frequently adopted [91–93]. However, no comparative study on the efficacy of different irrigation solutions has been carried out yet [56].

**Figure 1:**
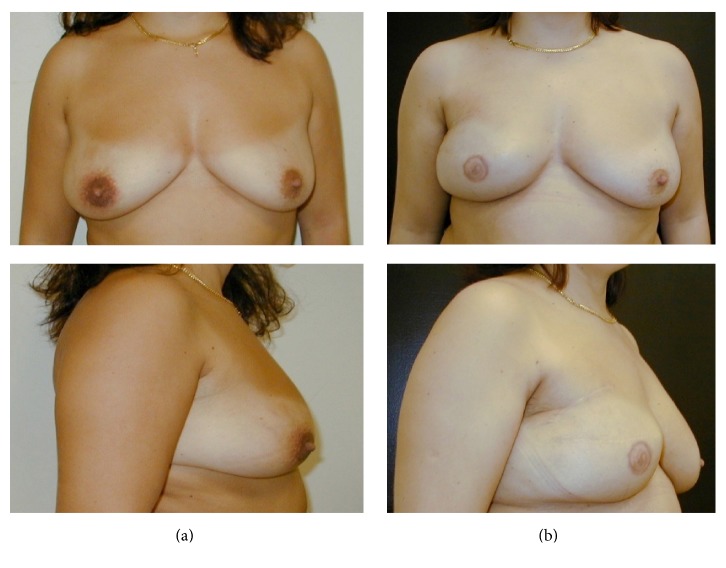
Preoperative view (a) and postoperative result (b) after right breast skin-sparing mastectomy with radial lateral incision and one-stage implant-based reconstruction after. Nipple-areola complex was reconstructed with local flap and tattooing. Matching surgery of the left breast was not required.

**Figure 2:**
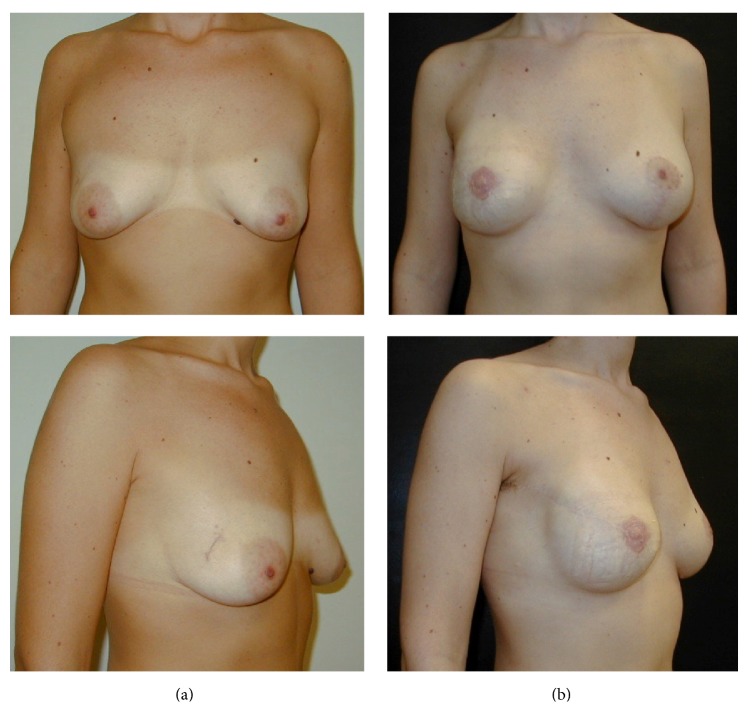
Preoperative view (a) and postoperative result (b) after right breast nipple-sparing mastectomy with radial lateral incision and one-stage implant-based reconstruction. Left breast augmentation with periareolar incision and vertical extension was also performed.

Two suction drains are usually positioned, one over the pectoralis major and under the mastectomy skin flap, and the other one at the level of the inframammary fold, coursing medially [57]. If the implant pocket is subcutaneous, only one suction drain in the inframammary fold can be positioned [58]. The skin is commonly closed in two layers [94] (Figures 1 and 2).

### 4.4. Postoperative Care

Intravenous prophylactic antibiotics are commonly given to patients undergoing direct-to-implant IBR 60 min or less prior to the time of incision [95]. Antibiotic administration should not be continued beyond the first 24 postoperative hours since there is no recommendation for prolonged postoperative antibiotics unless drainage is present [56, 96, 97]. In cases where a drain is still in place after 24 postoperative hours, the role of antibiotics is controversial, and surgeons should adhere to the hospital guidelines on antibiotic administration [98–100]. Drains should be left in place until the output is less than 20 mL/24 h, lowering the thresholds from the classic 30 mL/24 h [95]. Soft compression dressings in a nonconstricting surgical bra are used postoperatively to uniformly distribute very gentle pressure over the breast. It has been demonstrated that these measures can reduce both seroma and infection rates [95]. Postoperative pain is usually not long lasting and can be easily managed with painkillers. A supportive brassiere should be worn for the first postoperative month. Patients should avoid intense physical activity for the first 2-3 weeks.

## 5. Secondary Procedure

NAC reconstruction can be performed as early as the 2nd postoperative month by means of local flaps; tattooing is delayed till 6 weeks after NAC reconstruction [101]. Autologous fat grafting is a safe and effective secondary procedure after direct-to-implant IBR. Autologous fat grafting can ameliorate any residual contour deformities by correcting visible implant edges, asymmetry with the contralateral breast, and upper outer defects underneath the anterior axillary fold [102]. Furthermore, owing to the presence of a stem cell population, the so-called adipose-derived stem cells, fat grafts display regenerative potential and therapeutic effects that go beyond simple filling capability [103–107]. Adipose-derived stem cells can differentiate into multiple cell lineages and secrete paracrine factors [108–112]. Thus, angiogenesis and wound healing are strongly enhanced, leading to higher fat-graft survival as well as dermal and subcutaneous tissue regeneration [113–115]. Moreover, autologous fat grafting has been demonstrated to have positive effects on radiation-induced soft-tissue damage in reconstructed breasts [116, 117]. Fat grafts can thicken the subcutaneous tissue and improve the texture of the irradiated skin by enhancing its vascular supply [118].

## 6. Complications

Direct-to-implant IBR has the same postoperative complications as two-stage tissue expander/implant-based breast reconstructions [74]. However, two-stage breast reconstruction with submuscular implant pocket has traditionally been the reconstructive strategy of choice, given its demonstrated safety, reliability, and effectiveness [119–121]. Direct-to-implant IBR has recently gained popularity as a consequence of improvements in implant design and the advent of ADMs/synthetic meshes [17, 122–124].

Reports on the complication rates of direct-to-implant IBR are controversial, and these rates are supposed to be higher than those of tissue expander/implant-based reconstruction [19, 21, 22, 30]. Recently, Basta et al. [74] conducted the first head-to-head meta-analysis of the outcomes and complication rates of direct-to-implant IBR versus two-stage submuscular tissue expander/implant-based breast reconstruction. The pooled absolute incidence rates of infection (7.8% versus 7.4%), seroma (6.8% versus 7.1%), hematoma (4.3% versus 5.2%), and capsule contracture (13.5% versus 13.8%) did not significantly differ between direct-to-implant and two-stage tissue expander/implant-based reconstructions. However, the incidence rates of flap necrosis (8.6% versus 6.7%), reoperation (17.9% versus 13.8%), and implant loss (14.4% versus 8.7%) were significantly higher for direct-to-implant reconstructions. Unfortunately, there was no mention of whether the reconstructions were implant-based alone or ADM/mesh-assisted; therefore, no subgroup analysis was performed. Similarly, secondary procedure rates were not reported.

Jagsi et al. [65] retrospectively evaluated a series of 14,894 women undergoing either autologous reconstruction or direct-to-implant IBR with a mean follow-up of 2 years. Patients with autologous reconstruction reported higher wound complication rates (9.5% versus 4.4%) as well as higher infection rates (20.7% versus 20.5%) than patients who underwent implant-based reconstructions. Adjuvant radiation therapy was given to 35% of patients and was not associated with any infection in any surgical group. Among patients who received postoperative radiotherapy, the rate of implant removal was higher in the alloplastic breast reconstruction group (21.9% versus 13.1%), while the rate of fat necrosis was higher in the autologous reconstruction group (14.7% versus 8.7%).

Sbitany et al. [125] published a systematic review and meta-analysis of complications associated with ADM-assisted breast reconstruction compared with traditional implant-based musculofascial flaps. Skin-flap necrosis was the most common complication (10.9%), followed by seroma (6.9%), infection (5.7%), cellulitis (2%), and hematoma (1.3%). The rate of hematoma was 1.3% (95% CI, 0.6%–2.4%). Implant removal was necessary in 5.1% of cases. However, the rate of observed capsule contracture was very low (0.58%). ADM-assisted reconstruction was associated with a four times higher rate of seroma formation and nearly 3 times higher rates of infection and reconstruction failure than non-ADM-assisted breast reconstruction. Capsule contracture occurred in 0.58% of ADM-assisted breast reconstructions, while in the literature, the reported incidence of capsule contracture after breast reconstructions without the use of ADM ranges between 3% and 18% [126–129]. Ibrahim et al. [33] retrospectively analyzed 19,100 alloplastic breast reconstructions, of which 3301 were ADM-assisted. They reported no statistically significant difference in the overall complication rate between breast reconstruction with and without ADMs. Furthermore, they confirmed that a high body mass index, diabetes, history of smoking, radiotherapy, and steroid administration are associated with higher complication rates.

Salibian et al. [47] reviewed subcutaneous implant-based breast reconstructions with ADMs or meshes. The major complications (i.e., wide infection, complete NAC necrosis, complete flap necrosis, explantation, and Baker grade III/IV capsule contracture) were low in the majority of studies. Explantation (6%) was the most frequent complication, followed by seroma (4.9%), partial NAC necrosis (3.9%), wound healing problems (3.6%), and hematoma (2.4%). Direct-to-implant IBR and tissue expander/implant-based breast reconstruction had similar complication rates, even though statistical analysis could not be performed. However, interestingly, direct-to-implant IBR had higher explantation rates (6.0% versus 0%), while tissue expander/implant-based breast reconstruction had a higher rate of minor infection (16.0% versus 0%). Furthermore, subgroup analysis was performed between mesh- and ADM-assisted reconstructions. The most frequent complication of mesh-assisted reconstruction was minor infection (6.3%), followed by partial NAC necrosis (4.2%) and explantation (3.1%). ADM-assisted reconstructions were associated with higher complication rates: seroma formation, 8.9%; explantation, 6.7%; and partial NAC necrosis, 5%. The most common patient complaints after subcutaneous implant-based breast reconstruction were palpable implants (8.5%), rippling (4.7%), and visible implants (4.3%). Secondary procedures were needed in 21.4% of patients, where autologous fat grafting accounted for 11.9% of patients, and implant exchange was performed in 14.3% of patients [45, 130, 131].

## 7. Outcome

Direct-to-implant IBR has several potential advantages over traditional two-staged tissue expander/implant-based reconstruction such as avoidance of a second operation and no need for tissue expansion [74, 132, 133]. Indeed, final implant placement after expansion takes place in the 9th postoperative month on average. This span of time required to obtain the final result can be perceived as a significant burden to many patients. Direct-to-implant IBR implies a shorter time to reach the final reconstruction, which reduces the number of clinical visits and the sense of mutilation perceived, potentially improving patient quality of life [134, 135]. Nevertheless, direct-to-implant IBR has considerable wound issues such as mastectomy flap necrosis, which can vary from minor epidermolysis to full-thickness necrosis [74, 136]. When mastectomy flap necrosis occurs during expansion, partial deflation of the expander can allow surgical debridement and salvage reconstruction without expander removal. Conversely, direct-to-implant IBR more often requires implant exchange, which potentially compromises the final aesthetic outcome, lengthens recovery times, decreases the patient's quality of life, and delays the administration of adjuvant therapies, while increasing the economic costs to the patient and the healthcare system.

These aspects must be clearly discussed with patients in the preoperative setting, and the surgeon should highlight not only the possible shorter reconstructive course of direct-to-implant IBR but also its higher rate of reoperation and/or initial reconstructive failure. Intraoperative objective assessment tools such as real-time perfusion mapping assisted by SPY (Novadaq Technologies Inc., Bonita Springs, FL, USA) can lower the complication rate [89]. However, these devices are often expensive, time-consuming, and not readily available at all surgical centers [137].

Submuscular pockets provide additional coverage to implants [137]. However, muscle dissection can increase postoperative pain, and the submuscular location of the implant can result in action deformity and a less natural cosmetic outcome [138–141]. Furthermore, submuscular pockets can weaken even modest shoulder joint function, significantly impacting daily activities [139, 142, 143].

ADM-assisted reconstruction can reduce the operative time and speed postoperative recovery as a result of lower postoperative pain and donor-site morbidity [45]. Rapid return to work and prompt administration of adjuvant therapy when needed are further advantages. Moreover, the ADM/mesh-assisted wrapping technique is a muscle sparing-technique that can achieve good cosmetic outcome while preserving the pectoralis major muscle elevation and occurrence rate of other minor complications [144]. This approach is particularly suited for elderly patients where fast recovery and lower morbidity are mandatory for a better quality of life [58].

ADMs can cost between $2100 and $3400, depending on the size of the dermal sheet required [145]. However, it has been found that ADMs are a cost-effective therapeutic adjunct for breast reconstruction due to their better long-term aesthetic and clinical benefits [146, 147]. Furthermore, ADM-assisted reconstructions do not have significantly higher overall complication rates than non-ADM-assisted reconstructions but have lower long-term capsule contracture rates [33, 148, 149]. Given the uncertainties regarding the indications and contraindications, an algorithmic approach to aid decision-making with regard to the use of ADMs has been proposed [150, 151]. Indeed, judicious selection of candidates, careful evaluation of postmastectomy skin flaps, and consideration of possible risk factors have demonstrated the benefits of ADM-assisted breast reconstructions [57].

## 8. Conclusions

Direct-to-implant IBR is attractive given the good aesthetic outcomes, shared advantages with convention two-stage reconstruction, and patient-satisfaction rate achieved [152–154]. With the development of ADMs, a paradigm shift from conventional two-stage breast reconstruction to direct-to-implant one-stage IBR has been seen [135]. Nevertheless, the latter surgery has some drawbacks that we believe can be overcome by careful patient selection and strict adherence to surgical technique. However, larger comparative studies and better-defined selection criteria and outcomes reporting are needed to develop appropriate indications for performing successful direct-to-implant IBR.
